# Phosphorylation modulates secondary structure of intrinsically disorder regions in RNA polymerase II

**DOI:** 10.1016/j.jbc.2025.108533

**Published:** 2025-04-22

**Authors:** Wei Chen, Tatiana N. Laremore, Neela H. Yennawar, Scott A. Showalter

**Affiliations:** 1 Department of Chemistry, The Pennsylvania State University, University Park, Pennsylvania, USA; 2 Center for Eukaryotic Gene Regulation, Department of Biochemistry and Molecular Biology, The Pennsylvania State University, University Park, Pennsylvania, USA; 3 Huck Institutes of the Life Sciences, The Pennsylvania State University, University Park, Pennsylvania, USA

**Keywords:** intrinsically disordered proteins, NMR, phosphorylation, SAXS, transient secondary structures, transcription

## Abstract

The intrinsically disordered C-terminal domain (CTD) of RNA polymerase II contains tandem repeats with the consensus sequence YSPTSPS and coordinates transcription and cotranscriptional events through dynamic phosphorylation patterns. While it has been long hypothesized that phosphorylation induces structural changes in the CTD, a direct comparison of how different phosphorylation patterns modulate the CTD conformation has been limited. Here, we generated two distinct phosphorylation patterns in an essential *Drosophila* CTD region with the kinase Dyrk1a: one where Ser2 residues are primarily phosphorylated, mimicking the state near transcription termination, and a hyperphosphorylation state where most Ser2, Ser5, and Thr residues are phosphorylated, expanding on our work on Ser5 phosphorylation, which mimics early transcription elongation. Using ^13^C Direct-Detect NMR, we show that the CTD tends to form transient beta strands and beta turns, which are altered differently by Ser2 and Ser5 phosphorylation. Small-angle X-ray scattering revealed no significant changes in the CTD global dimensions even at high phosphorylation levels, contradicting the common assumption of phosphorylation-induced chain expansion. Our findings support a transient beta model in which unphosphorylated CTD adopts transient beta strands at Ser2 during transcription preinitiation. These transient structures are disrupted by Ser5 phosphorylation in early elongation, and later restored by Ser2 phosphorylation near termination for recruiting beta turn-recognizing termination factors.

The intrinsically disordered C-terminal domain (CTD) of RNA polymerase II regulates the transcription cycle and cotranscriptional RNA processing through its dynamic phosphorylation patterns (1, 2, 3, 4). The CTD comprises tandem repeats of the consensus heptad sequence Y_1_S_2_P_3_T_4_S_5_P_6_S_7_, with the number of repeats varying from 26 in yeast to 52 in vertebrates (2). During transcription, CTD heptads undergo constant phosphorylation and dephosphorylation, creating distinct phosphorylation patterns—collectively known as the CTD code (5). These phosphorylation patterns specify the progress of transcription and recruit various regulators including the Mediator complex, mRNA processing enzymes, and termination factors (2, 6, 7, 8, 9). Genome-wide analyses revealed a universal CTD code across genes (1), with phosphorylation at Ser5 and Ser2 being the most abundant (10, 11). Phospho-Ser5 (pSer5) levels peak early in transcription elongation and decline progressively, while phospho-Ser2 (pSer2) levels increase during elongation and peak near termination (1, 12). Phospho-Thr4 (pThr4), although less abundant (10, 11), is also enriched near termination (13). It has been proposed that phosphorylation patterns modulate the conformation of the CTD in distinct ways to mediate interactions with various coregulators for cotranscriptional RNA processing (14).

Structural characterization of the CTD has been challenging due to its intrinsically disordered nature. Studies on synthetic CTD peptides with NMR and CD spectroscopy revealed its lack of structure and a weak propensity toward beta turn (<10%) and polyproline II (<15%) (15, 16, 17). Beta turns form around Ser2 and Ser5 and are stabilized in trifluoroethanol or in circular peptides (16, 18). Because of the low population and the transient nature of secondary structures in the CTD, structural changes associated with phosphorylation are also difficult to probe. NMR and CD spectroscopy were not able to detect stable beta turns in CTD phospho-peptides (17, 19). However, when the CTD is bound to binding partners, its beta turns become stabilized and were captured in a number of crystal structures in both unphosphorylated and phosphorylated states (7, 20, 21, 22, 23, 24, 25, 26, 27). While intrinsically disordered proteins (IDPs) with alpha helical propensities and their roles in molecular recognition have been extensively studied (28, 29, 30), literature on IDPs with transient beta strands and beta turns remains limited. The CTD provides a critical example for exploring the structural and functional roles of transient secondary structures beyond alpha helices.

Phosphorylation of the CTD has been assumed to expand the global conformation of the CTD as a result of electrostatic repulsion between negatively charged phosphate groups (3, 4, 14, 16, 31). Similar expansion is expected for other IDPs that undergo multisite phosphorylation (32). This assumption is supported by studies using electrophoresis, gel filtration chromatography, sucrose gradient ultracentrifugation, and limited proteolysis (33, 34, 35), where unphosphorylated and phosphorylated CTD behave differently. However, small-angle X-ray scattering (SAXS) studies on a 12-heptad region of *Drosophila* CTD and maltose binding protein (MBP)-tagged full-length *Drosophila* CTD suggest minimal changes in the radius of gyration (Rg) upon phosphorylation (34, 36).

How different phosphorylation patterns impact the CTD conformation throughout transcription remains unclear. Although several NMR and computational studies have explored the conformational properties of short CTD peptides (typically 1–3 heptads) with different phosphorylation states (19, 31, 37), structural characterization of physiologically relevant, longer phosphorylated CTDs remains limited (34, 36). In our previous work, we addressed this gap by focusing on the CTD of *Drosophila melanogaster* (34, 36). The *Drosophila* CTD consists of 42 heptad repeats, only two of which follow the exact consensus sequence Y_1_S_2_P_3_T_4_S_5_P_6_S_7_, allowing for residue-specific characterization with NMR and mass spectrometry (MS). Previously, we characterized a 12-heptad *Drosophila* CTD region essential for viability, referred to as CTD2′, in unphosphorylated and pSer5 states, and discovered that pSer5 promotes *cis*-Pro6 formation in heptads containing Asn7 (36). This work provides insights into the structural consequences of a phosphorylation state similar to the state in early elongation, but structural effects of other important phosphorylation marks, particularly pSer2, remain largely unexplored.

Here, we investigate the conformational properties of CTD2′ with two new phosphorylation patterns created by the kinase Dyrk1a using ^13^C Direct-Detect NMR and SAXS. The phosphorylation patterns include one where Ser2 are primarily phosphorylated, mimicking the state near transcription termination, as well as a hyperphosphorylation state, where all Ser2, Ser5, as well as nonconsensus Thr residues are phosphorylated. These additional phosphorylation patterns allow for a direct comparison of structural changes induced by pSer5 *versus* pSer2, providing insights into the structural consequences of changing phosphorylation patterns during transcription. We found that the global dimensions of CTD2′ remain unchanged even when 20% of the residues are phosphorylated. Phosphorylation at Ser5 is associated with a substantial reduction in beta-strand and turn propensities, whereas phosphorylation at Ser2 results in a comparatively minor decrease. Our data support a transient beta model in which transient beta turns in unphosphorylated CTD preinitiation are disrupted by Ser5 phosphorylation in early elongation, and are restored by Ser2 phosphorylation near termination, facilitating termination factor recruitment.

## Results

### Dyrk1a preferentially phosphorylates Ser2 and Thr4 in CTD2′

In our previous study, we phosphorylated CTD2′ using the positive transcription elongation factor b (P-TEFb), which generated up to 12 phosphorylation marks, predominantly at Ser5 residues (36, 38). To investigate how different phosphorylation patterns affect the conformational properties of the CTD, we expanded our CTD kinase toolkit to include Dyrk1a (dual specificity tyrosine-phosphorylation-regulated kinase 1A), a kinase associated with Down syndrome that has been reported to phosphorylate Ser2 specifically *in vitro* (39, 40, 41). To generate a distinct phosphorylation pattern from that generated by P-TEFb, we performed *in vitro* phosphorylation of CTD2′ with Dyrk1a and analyzed the results using MS and NMR.

**Figure 1:**
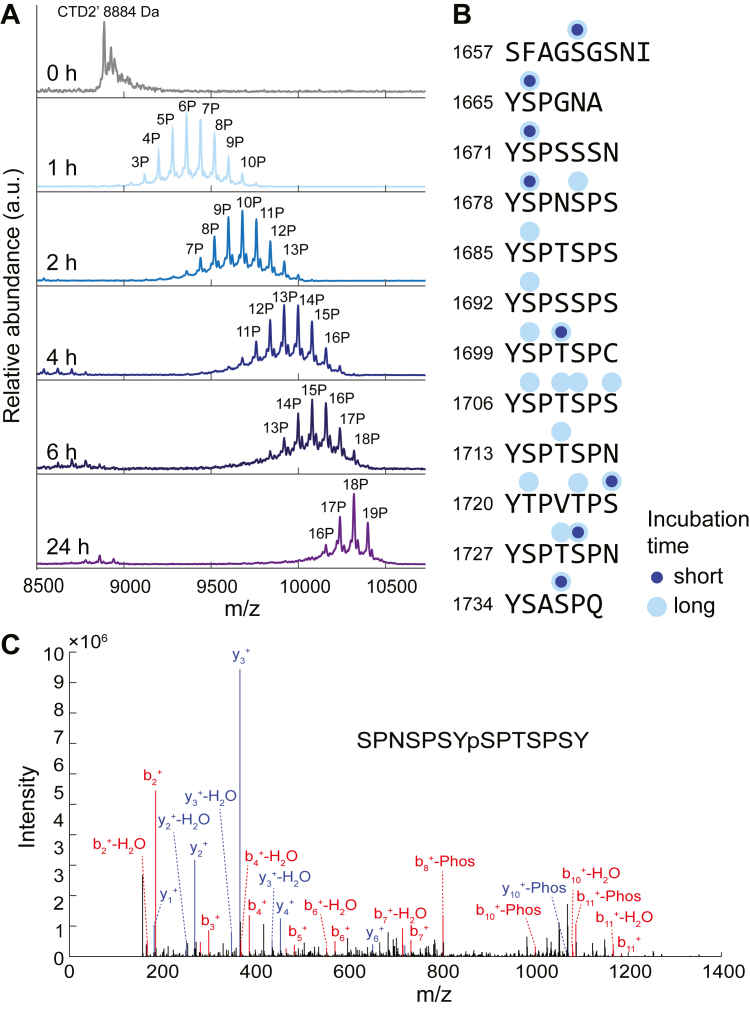
**Phosphorylation of CTD2′ by Dyrk1a characterized with mass spectrometry.***A*, MALDI-TOF mass spectra of intact CTD2′ after phosphorylation by Dyrk1a for varying incubation times. *B*, phosphorylation sites identified by nano-LC MS^2^ analysis of Dyrk1a phosphorylated CTD2′ with different incubation times. *C*, representative peptide spectrum from nano-LC MS^2^ analysis of Dyrk1a phosphorylated CTD2′. CTD, C-terminal domain; nano-LC MS^2^, nano-liquid chromatography-tandem mass spectrometry.

To quantify the degree of phosphorylation, we performed MALDI-TOF MS experiments on intact CTD2′ phosphorylated by Dyrk1a for various incubation times (Fig. 1*A*). Since CTD2′ contains 12 heptad repeats, 10 of which include a Ser2 residue, complete phosphorylation at every Ser2 but not at other positions would yield a mass shift of +800. However, we detected more than 10 phosphorylation events after 1 h of incubation, indicating that Dyrk1a is not strictly specific to Ser2 in CTD2′. Incubation for 24 h resulted in up to 19 phosphorylation events with no further phosphorylation observed after 48 h (Fig. S1*A*).

The high degree of phosphorylation suggested that Dyrk1a also phosphorylated additional Ser/Thr residues. Previous studies reporting the *in vitro* specificity of Dyrk1a for Ser2 were conducted using CTD constructs with only consensus heptads (YSPTSPS) (40). To investigate the specificity of Dyrk1a toward CTD2′, which contains only two consensus heptads among its 12 repeats, we performed liquid chromatography-tandem mass spectrometry (nano-LC MS^2^) on chymotrypsin-digested phosphorylated CTD2′. Phosphorylation sites were identified for two phosphorylated CTD2′ samples incubated with Dyrk1a for different durations, corresponding to an average of 6 and 12 phosphorylation marks, respectively as quantified by MALDI-TOF (Fig. S1, *B* and *C*). Both data sets had 100% sequence coverage for nano-LC MS^2^ (Fig. S1, *B* and *C*), and showed that Dyrk1a preferentially phosphorylated Ser2 over Ser5 or Ser7 (Fig. 1, *B* and *C*), although phosphorylation at Ser5 and Ser7 was also observed. Interestingly, Thr residues were phosphorylated to a significant degree, which has not been previously reported for Dyrk1a (39, 40). In the first consensus heptad (residues 1685–1691), only Ser2 was phosphorylated, whereas in the second consensus heptad (residues 1706–1712), all Ser and Thr were phosphorylated.

**Figure 2:**
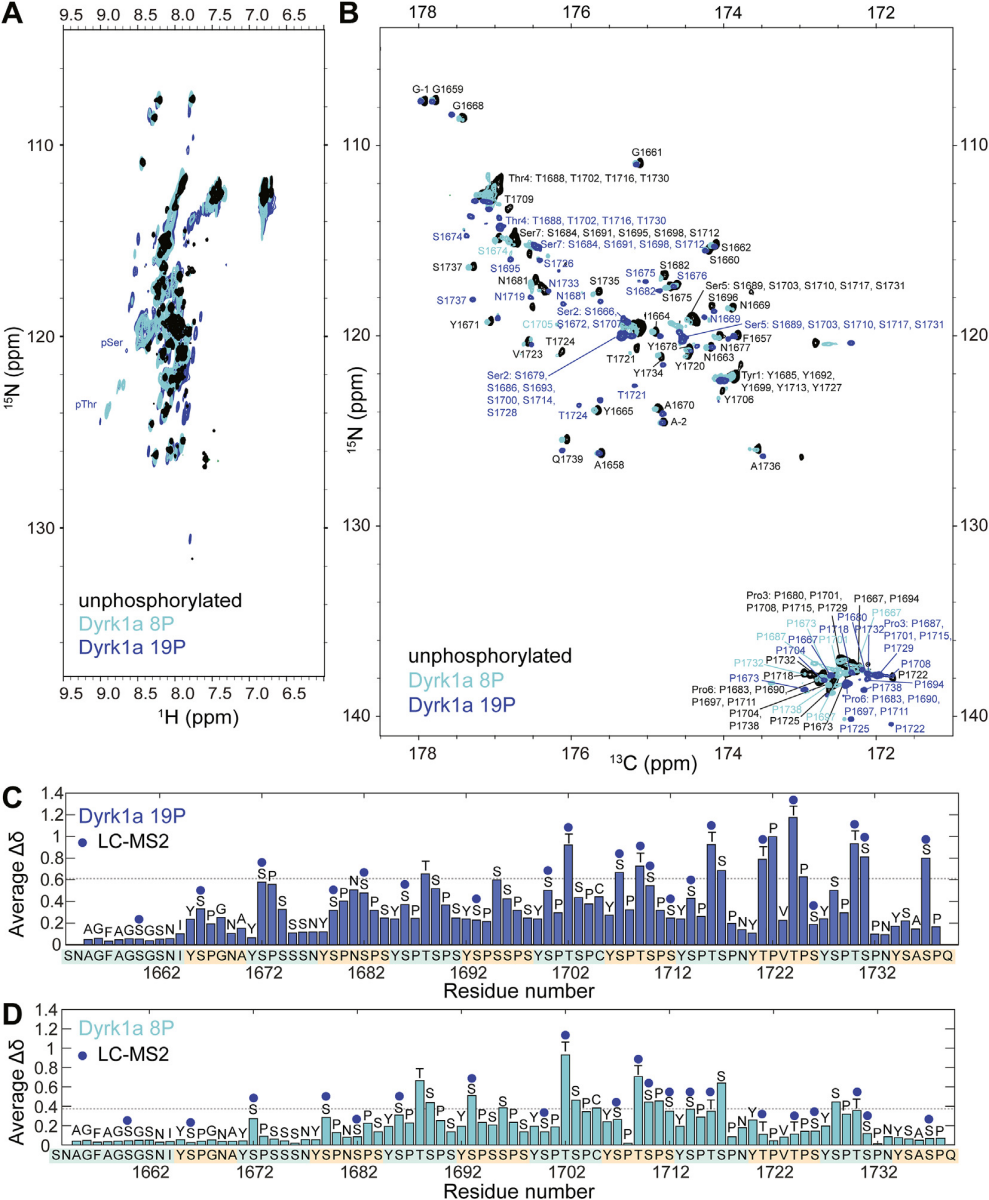
**Phosphorylation of CTD2′ by Dyrk1a characterized with NMR.***A,*^1^H, ^15^N-HSQC spectra and *B,*^13^C, ^15^N-CON spectra of unphosphorylated CTD2′ and Dyrk1a phosphorylated CTD2′ with 8 and 19 phosphorylation marks (8P and 19P). *C*, chemical shift perturbation (CSP) analysis for CTD2′ 19P with phosphorylation at SP/TP sites. *D*, CSP analysis of CTD2′ 8P shows an enrichment of pSer2. *Dotted lines* indicate one standard deviation above the mean. CTD, C-terminal domain.

To cross-examine the phosphorylation sites identified by MS, we conducted NMR analysis on Dyrk1a phosphorylated CTD2′ samples containing an average of eight phosphorylation marks and a fully phosphorylated state containing 19 marks (Fig. S2*A*). These samples represent two distinct states: one where, each heptad repeat contains approximately a single phosphorylated residue, and a hyperphosphorylated state where all Dyrk1a target sites are modified. The ^1^H, ^15^N-HSQC spectra of all CTD2′ samples exhibited severe signal overlap, particularly in the ^1^H dimension (Fig. 2*A*), which is common for IDPs. This lack of spectral dispersion was especially pronounced for CTD2′ due to its highly repetitive sequence. Similar crowded ^1^H, ^15^N-HSQC spectra were observed in our previous studies of P-TEFb phosphorylated CTD2′ (36). While downfield shifts in the ^1^H dimension for Ser and Thr residues upon phosphorylation were observed (Fig. 2*A*), a complete residue-level assignment for CTD2′ based on ^1^H, ^15^N-HSQC was not feasible due to signal overlap. Additionally, the abundance of Pro residues in CTD2′, which lack amide protons and are therefore invisible in ^1^H, ^15^N-HSQC, further complicated conventional proton-detected NMR assignment methods. To overcome these challenges, we utilized ^13^C Direct-Detect NMR, as in our previous CTD2′ studies (36). We acquired two-dimensional ^13^C, ^15^N-CON spectra for unphosphorylated and Dyrk1a phosphorylated CTD2′ (Figs. 2*B*), in which each peak corresponds to the correlation between the amide nitrogen of residue *i* and carbonyl carbon of residue *i - 1*. The CON spectra demonstrated remarkable spectral dispersion for all CTD2′ samples and allowed for the direct detection of Pro residues. Using a suite of two-dimensional and three-dimensional ^13^C Direct-Detect NMR experiments (42), we successfully assigned the CTD2′ spectra (Figs. 2*B*, S3). As an example, we include a representative backbone assignment example illustrating the connectivity from Y1692 to T1702 (YSPSSPSYSPT) (Fig. S4).

We then examined the chemical shift perturbation (CSP) caused by phosphorylation. For the hyperphosphorylated CTD2′ sample containing 19 phosphorylation marks (referred to as 19P), the number of marks matched the number of SP/TP motifs, enabling phosphorylation assignment. The Ser and Thr residues showing significant CSPs were consistent with the phosphorylation sites identified by nano-LC MS^2^ (Fig. 2*C*). Phosphorylation also induced shifts in the C_β_ resonances for T1721 and T1724, the only two TP motifs in CTD2′, whereas such shifts were less pronounced or not observed for Ser residues (Fig. S5). This hyperphosphorylation pattern was enriched in pSer and pThr residues with SP/TP motifs, leading to increased levels of pSer2, pSer5, and pThr. The CON spectrum of the CTD2′ sample with an average of eight phosphorylation marks (referred to as 8P) displayed multiple sets of peaks. Some peaks overlapped with those from the unphosphorylated and hyperphosphorylated CTD2′, while others were unique (Figs. S2*B*, S3), indicating a mixture of phosphorylation states. CSP analysis of the most intense peaks in the CTD2′ 8P spectrum, which represent the major species in the mixture, showed fewer phosphorylation sites compared to the 19P sample as expected, with an enrichment of pSer2 (Fig. 2*D*). Thr4 also exhibited high CSPs; however, C_β_ resonance shifts for Thr4 were not observed in CCCON strips (Fig. S5), as seen for T1724 (TP motifs) in a minor state. Notably, this phosphorylation pattern, enriched in pSer2 and possibly pThr4, resembles the *in vivo* CTD phosphorylation pattern near transcription termination, in contrast to the pattern generated by P-TEFb, which mirrors the *in vivo* phosphorylation pattern near the transcription start site.

### Pro residues remain in trans states in Dyrk1a phosphorylated CTD2′

**Figure 3:**
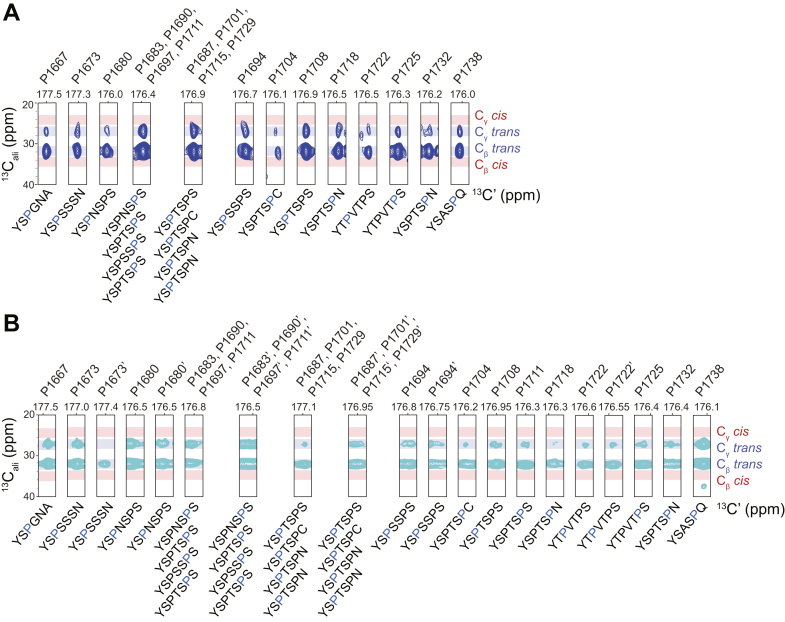
**Analysis of proline *cis-trans* conformations in Dyrk1a phosphorylated CTD2′.***A*, C_β_ and C_γ_ chemical shifts of individual Pro residues from 3D CCCON spectra of hyperphosphorylated CTD2′ (19P). *B*, C_β_ and C_γ_ chemical shifts of individual Pro residues from partially phosphorylated CTD2′ (8P). *Blue* and *red* areas represent the chemical shift ranges for *trans* and *cis* Pro conformations, respectively. For partially phosphorylated CTD2′ (8P), multiple sets of peaks were observed for certain residues, with the additional set labeled with an apostrophe (‘). CTD, C-terminal domain.

We previously showed that phosphorylation at Ser5 in CTD2′ promotes the formation of *cis*-Pro6 in the nonconsensus heptad Y_1_S_2_P_3_T_4_S_5_P_6_N_7_. Since Dyrk1-phosphorylated CTD2′ contains a heptad with the sequence Y_1_S_2_P_3_N_4_S_5_P_6_S_7_, where Ser2 (S1679) is in the context of SPN, we examined whether pSer2 in the SPN motif can also induce *cis*-Pro formation in CTD2′ 8P. Additionally, we investigated the Pro *cis/trans* state in CTD2′ 19P, where Ser and Thr adjacent to Pro residues, including both Ser2 and Ser5, were phosphorylated. The *cis/trans* state of Pro residues was assessed using the C_β_ and C_γ_ chemical shifts from three-dimensional CCCON spectra (Fig. 3). Unlike P-TEFb phosphorylated CTD2′, we did not observe an enrichment of *cis*-Pro3 in the SPN sequence context, suggesting that pSer-induced proline isomerization depends on sequence context beyond these three residues. In CTD2′ 19P, we also did not find enrichment for *cis*-Pro3 or *cis*-Pro6, suggesting that the propensity *cis*-Pro induced by Ser5 is counteracted when the Ser2 and/or other Thr residues are also phosphorylated.

### Phosphorylation patterns modulate secondary structure propensity in CTD2′

**Figure 4:**
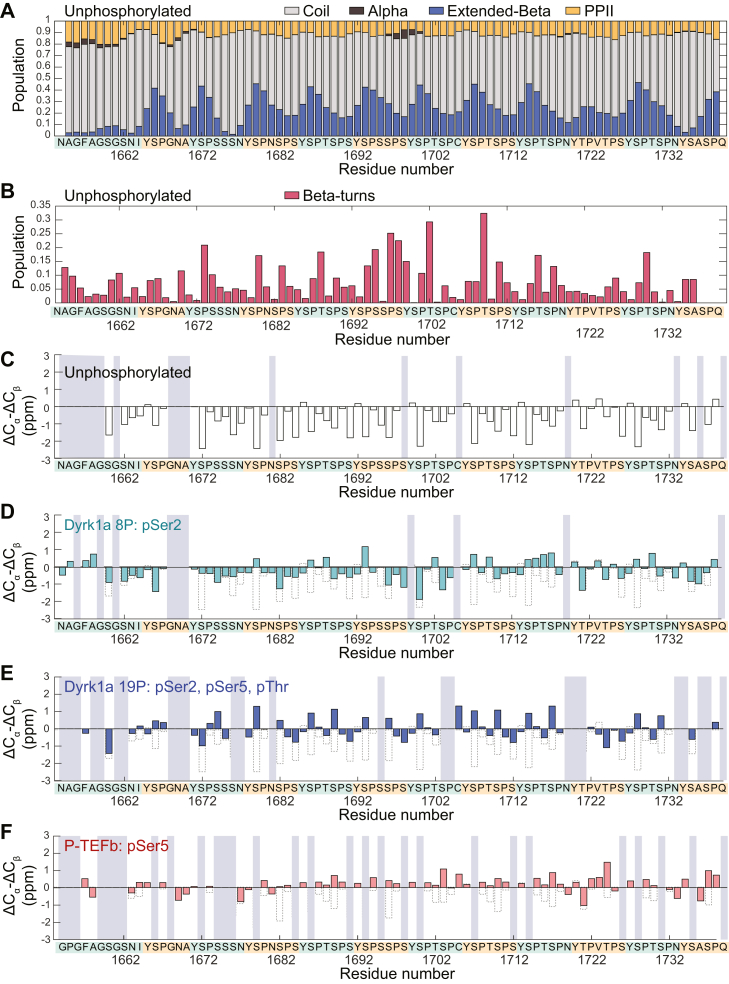
**Secondary structure propensity for CTD2′ with different phosphorylation patterns.***A*, populations of coil, alpha helix, extended beta strand, and polyproline II (PPII) for unphosphorylated CTD2′ calculated from backbone and side chain chemical shifts using the δ2D program. *B*, population of beta turns in unphosphorylated CTD2′ calculated using the MICS program. *C*, secondary chemical shifts for unphosphorylated CTD2′, *D,* secondary chemical shifts for Dyrk1a phosphorylate CTD2′ (8P), in which Ser2 residues are primarily phosphorylated. *E*, secondary chemical shifts for Dyrk1a phosphorylated CTD2′ (19P), in which Ser2, Ser5, and Thr residues are phosphorylated. *F*, secondary chemical shifts for P-TEFb phosphorylated CTD2′, in which Ser5 residues are phosphorylated. Residues without available C_α_ and/or C_β_ chemical shift values are indicated by gray areas. Secondary chemical shifts for unphosphorylated CTD2′ are overlaid to those for phosphorylated CTD2' as dotted bars. CTD, C-terminal domain; MICS, motif identification from chemical shifts; P-TEFb, positive transcription elongation factor b.

To explore how different phosphorylation patterns influence the transient secondary structure of CTD2′, we first calculated secondary structure populations using C_α_, C_β_, and backbone chemical shifts for unphosphorylated CTD2′ with the δ2D program (43), which has been widely used for IDPs (Fig. 4*A*). Overall, CTD2′ adopts a coil conformation with a 23.1% extended beta strand population. Particularly, beta strand populations peak periodically at Ser2 (40%). Since δ2D does not predict beta turn populations, we used the motif identification from chemical shifts (MICS) program to estimate beta turn propensity (44). While MICS was originally trained on databases of structured proteins, it provided consistent prediction for CTD2′ with a predominantly structural disorder and a significant beta strand propensity (Fig. S6). Remarkably, it identified beta turn populations at Pro3 and Thr4 across most heptads (Figs. 4*B*, S6), consistent with positions in NOE-based NMR studies (16, 18) and crystal structures of CTD peptides bound to Pcf11 (7). The results of δ2D and MICS align with a structural model where Tyr1 and Ser2 form transient beta strands, followed by a turn at Pro3 and Thr4.

To compare the secondary structure propensity across different phosphorylation patterns, we calculated deviations of C_α_ and C_β_ chemical shifts from their expected random coil values. Secondary chemical shifts (45, 46, 47), ΔC_α_-ΔC_β_, were calculated for unphosphorylated CTD2′, two Dyrk1a phosphorylated CTD2′ samples, and previously published P-TEFb phosphorylated CTD2′ (36). Positive ΔC_α_-ΔC_β_ values indicate a tendency toward alpha helix, while negative values suggest a propensity toward beta strand or extended structures. Consistent with δ2D, MICS, and our previous work (36), unphosphorylated CTD2′ shows negative ΔC_α_-ΔC_β_ values in general, indicating a disordered state with a preference for extended beta strand (Fig. 4*C*). For unphosphorylated and phosphorylated samples, random coil chemical shift values were determined using the database published by the Kragelund group (47). For the Dyrk1a-phosphorylated CTD2′ 8P, although exact phosphorylation sites cannot be definitively assigned from the NMR spectra, a general enrichment of pSer2 was observed. We used several possible phosphorylation assignments to generate four different sets of random coil chemical shift references, all of which produced similar results in terms of the general negative secondary chemical shifts (Fig. S7). Compared to unphosphorylated CTD2′, the beta propensity of Dyrk1a-phosphorylated CTD2′ 8P is reduced but largely retained—especially when compared to the other two phosphorylation patterns (Fig. 4*D*). In hyperphosphorylated CTD2′ 19P, where Ser2, Ser5, and Thr residues are phosphorylated, the beta propensity greatly decreases, as indicated by near-zero ΔC_α_–ΔC_β_ values across all heptads (Fig. 4*E*). Interestingly, P-TEFb phosphorylated CTD2′ (Fig. 4*F*), which contains an average of nine phosphorylation marks at Ser5, showed a similar degree of reduction in beta propensity as hyperphosphorylated CTD2′ 19P, which contains about twice as many phosphorylation marks. This suggests that the secondary structure propensity in CTD2′ is modulated by specific locations and patterning of phosphorylated residues rather than by the total number of phosphorylation marks. The beta propensity in unphosphorylated CTD2′ is retained when Ser2 residues become phosphorylated, but is disrupted when Ser5 residues are further phosphorylated. The beta propensity is also disrupted when phosphorylation is only at Ser5.

### CTD2′ retains its global dimensions regardless of phosphorylation states

It has been widely proposed that multisite phosphorylation of IDPs can lead to chain expansion due to electrostatic repulsion between negatively charged phosphate groups (32). In our previous work, we showed that phosphorylation of CTD2′ by P-TEFb, which introduces an average of nine phosphorylation marks (∼10% of the residues), did not result in changes in the radius of gyration (R_g_) and pairwise distance distribution, P(r), as determined by SAXS (36). We attributed this to Debye screening from the ions in solution. Here, we further investigated whether a higher degree of phosphorylation by Dyrk1a, which added more closely spaced phosphate groups on Ser and Thr residues, could cause chain expansion due to expected shorter distances between charges.

**Figure 5:**
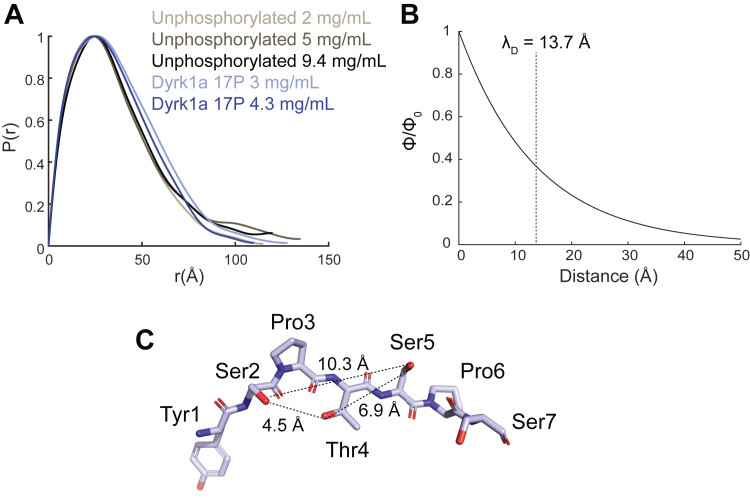
**SAXS analysis of global dimensions and Debye screening in CTD2′.***A*, representative pair-wise distance distributions, P(r), for unphosphorylated CTD2′ (*black*) and Dyrk1a phosphorylated CTD2′ 17P (*blue*). *B*, Debye screening of the electric potential for the SAXS buffer condition with a Debye length (λ_D_) of 13.7 Å. *C*, distances between Ser2, Thr4, and Ser5 residues in an extended conformation. CTD, C-terminal domain; SAXS, small-angle X-ray scattering.

We first carried out size-exclusion chromatography with multiangle light scattering (SEC-MALS) to confirm that CTD2′ was monomeric (Fig. S8), with a hydrodynamic radius (R_h_) of 16.12 ± 0.28 Å. We then performed SAXS on unphosphorylated and Dyrk1a phosphorylated CTD2′ containing an average of 17 phosphorylation marks (∼20% of the residues) (Fig. S9, Table S1). The R_g_ values calculated using the Guinier approximation are 27.99 ± 0.28 Å for unphosphorylated and 28.24 ± 0.25 Å for Dyrk1a phosphorylated CTD2′, consistent with our previously reported values (28.0 ± 0.7 for unphosphorylated and 28.3 ± 0.3 for P-TEFb-phosphorylated CTD2′). The R_g_/R_h_ ratio is typically 3/5 (∼0.775) for globular proteins, 1.24 for ideal Gaussian chains, and 1.56 for excluded volume chains (48). For CTD2′, the R_g_/R_h_ ratio is 1.736, indicating a more extended conformation compared to excluded volume chains. According to the polymer scaling law parametrized for IDPs (49), *R*_*g*_ = *R*_*0*_*N*^*ν*^, in which *R*_*0*_ and *ν* are 2.54 and 0.522 respectively, an IDP with the same number of residues as CTD2′ would have an R_g_ of 26.14 Å. This suggests that CTD2′ is slightly more extended than typical IDPs. In addition to the similarity in R_g_ values between unphosphorylated and highly phosphorylated CTD2′, there were no significant differences in the P(r) plots between the two states (Fig. 5*A*).

To evaluate whether Debye screening plays a major role in maintaining the chain dimension of highly phosphorylated CTD2′, we calculated the screening effect of electric potential using the buffer conditions from our SAXS experiments (Fig. 5*B*). The characteristic Debye screening length (λ_D_), defined as $\lambda_{D}=\frac{1}{\sqrt{4\pi l_{B}\sum_{i}\rho_{i}z_{i}^{2}}}$, where *l*_*B*_ is the Bjerrum length (7 Å), was calculated as 13.7 Å for our buffer containing 50 mM KCl (50). The Debye screening effect decays exponentially as the distance increases (Fig. 5*B*). To estimate the distances between phosphorylated residues in CTD2′, we measured the distances between side chain oxygen atoms in the residue pairs, Ser2-Ser5, Ser2-Thr4, and Thr4-Ser5, in a completely extended conformation (Fig. 5*C*). The measured distances were 10.3 Å, 4.5 Å, and 6.9 Å, respectively. Since CTD2′ is more compact than an extended coil, as indicated by SAXS, the actual distances between phosphorylated residues may be shorter than those measured in an extended conformation. Based on the above estimation, electrostatic interactions between phosphorylated residues in Dyrk1a phosphorylated CTD2′ do not seem to be completely screened out. This suggests that besides Debye screening, local conformational changes, as such changes in secondary structure propensity indicated by NMR, also contribute to maintaining the overall size and shape of CTD2′ upon phosphorylation.

## Discussion

The CTD of RNA polymerase II undergoes dynamic phosphorylation throughout the transcription cycle and recruits coregulators that recognize specific phosphorylation. It has long been suggested that phosphorylation causes the CTD to adopt a more extended conformation due to charge repulsion between phosphate groups (3, 4, 14). This hypothesis has been supported by earlier observations of differences between unphosphorylated and phosphorylated CTDs in gel-filtration chromatography and sucrose gradient ultracentrifugation (33), as well as a recent computational study on a 44-residue human CTD region (31). However, our previous SAXS studies on CTD2′ and the full-length *Drosophila* CTD phosphorylated at Ser5 by P-TEFb showed only modest or no changes in R_g_ (34, 36). Here, we further demonstrated that CTD2′ retains its global conformational compactness even when 20% of the total residues are phosphorylated This suggests that the CTD retains its global dimensions during the dynamic phosphorylation and dephosphorylation process in transcription, where typically ∼5% of residues are phosphorylated at a given time (10, 11).

Comparison of R_g_ values for CTD2′ (28 Å) and average IDPs with the same number of residues (24–26 Å) (51, 52) suggests that although the CTD has been described as “compact” compared to a fully extended chain, which is not thermodynamically favored, it is slightly more extended than average IDPs. Debye screening plays a role in physiological conditions (λ_D_ is ∼ 7 Å) in screening out most charge interactions. However, our 20% phosphorylated CTD2′ with closely spaced phosphate groups that can have separations smaller than the Debye length suggests that the conservation of global dimension may be an inherent property coded in the amino acid sequence of the CTD. Possible conformational changes that counteract the effect of charge repulsion include compaction at the scale of 1 to 2 heptads as a result of beta turn formation upon phosphorylation and coordination of phosphate groups with counterions in the buffer, both of which have been observed in computational studies (31, 37). The conformational rigidity of Pro residues, as well as their *cis-trans* isomerization coupled with phosphorylation, may also contribute to offsetting the structural impact of charge repulsion. From the perspective of function, the amino acid sequence of the CTD may be evolved for it to retain the global dimension in various phosphorylated states, so that the accessibility remains the same for unphosphorylated and differently phosphorylated CTD to bind to coregulators.

Early NMR studies on short synthetic CTD peptides showed that the CTD is highly flexible with a tendency to form beta-turns (15, 16). NMR secondary structure propensity analyses on long CTD constructs from our group and others are consistent with this view, showing that the CTD adopts a conformational ensemble with a beta-turn propensity around Ser2 (36, 53). It has been proposed that the CTD can adopt different conformations depending on specific phosphorylation patterns (14). Specifically, a beta-spiral model has been proposed (7, 14), in which the unphosphorylated CTD adopts a compact conformation where each heptad forms a beta turn, and these turns are stacked together in a spiral-like arrangement, which is opened up by Ser5 phosphorylation but not Ser2 phosphorylation (14). Our SAXS data suggest that the CTD retains its global compactness even when highly phosphorylated. Moreover, the CTD remains disordered across all phosphorylation states and does not adopt the stable secondary structures required for building a beta spiral. CD studies on the CTD were unable to detect population changes in beta-turns associated with phosphorylation (17, 19). With NMR chemical shifts, we are able to detect a significant degree of beta structural propensity in unphosphorylated CTD2′, which is disrupted by pSer5 but largely retained by pSer2. Interestingly, when both Ser5 and Ser2 are phosphorylated, the beta structural propensity is also lost. Our previous work showed that pSer5 promotes *cis*-Pro6 formation in nonconsensus heptads containing Asn7 (36). We note that this effect would not be unique to *Drosophila* because a similar cluster of heptads containing Asn7 is found in mammalian CTDs, including mouse and human ones. A computational study on single CTD heptads showed that *cis*-Pro6 inhibits beta turn formation (37), suggesting *cis*-Pro6 also plays a role in the lack of beta structure propensity for CTD2′ with pSer5.

**Figure 6:**
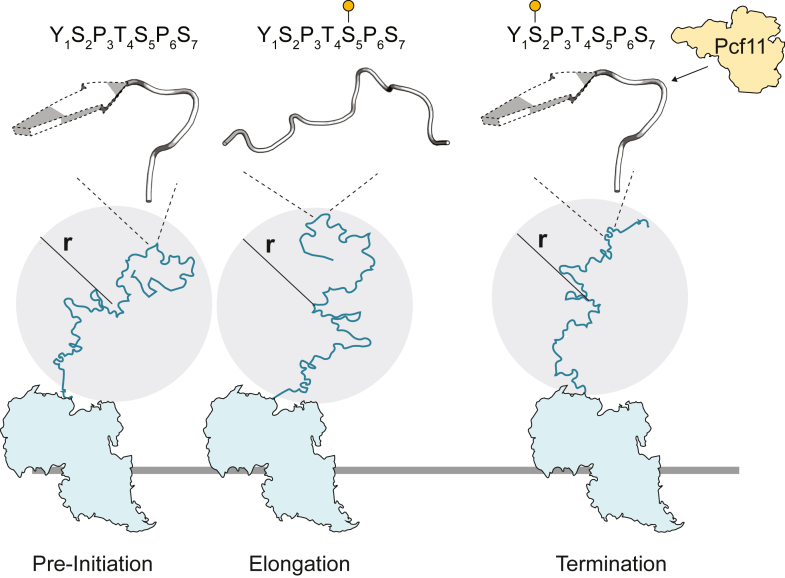
**A transient beta model for CTD conformational modulation by phosphorylation.** In the transcription preinitiation state, the unphosphorylated CTD adopts a flexible conformational ensemble with a 20% to 40% propensity to form transient beta strands and turns around Ser2. As transcription progresses, the CTD becomes phosphorylated primarily at Ser5 near the transcription start site, which disrupts the beta turns. During elongation, the decrease of pSer5 level combined with the increase of pSer2 level restores the beta propensity, which is crucial for binding to the termination factor Pcf11. Despite the changing phosphorylation patterns, the global dimensions of the CTD, as measured by r, remain conserved throughout transcription. This conservation of global size ensures accessibility to binding partners at different transcription stages, while changes in transient local structures modulate specific interactions with coregulators. CTD, C-terminal domain.

Our NMR and SAXS data support a transient beta model in which the CTD retains its overall compactness (Fig. 6), which is more extended than typical IDPs, potentially to allow accessibility to coregulators throughout the transcription cycle, including interactions with the Mediator in the unphosphorylated state for transcription preinitiation, as well as with various mRNA processing factors in differently phosphorylated states during elongation and near termination. While the global dimensions are conserved, the changing phosphorylation patterns modulate the transient local structures of the CTD. Phosphorylation at Ser5, which is prevalent near the transcription start site, decreases the beta propensity and promotes *cis*-Pro6 formation for Asn7-containing heptads. As transcription proceeds and the level of pSer5 decreases while pSer2 increases during elongation, the beta propensity is restored and the *cis*-Pro level decreases, facilitating the recognition of beta turns and *trans*-Pro by the termination factor Pcf11 (7, 19). Notably, Pcf11 does not directly interact with the phosphate group on Ser2 but rather binds to the beta turn (7, 19). Our NMR data of the hyperphosphorylated CTD reveals that in the presence of pSer5, the beta conformational propensity remains low, despite Ser2 phosphorylation, suggesting that pSer5, in addition to pTyr1 (54), may contribute to preventing premature termination by Pcf11 binding. The beta conformation becomes sufficiently populated to recruit Pcf11 near the polyadenylation site, where Ser5 residues are mostly dephosphorylated.

## Conclusion

Our results show that, contrary to assumptions about phosphorylation-induced chain expansion, the CTD retains its overall compactness even in highly phosphorylated states. While the global dimensions are conversed, transient local structures are modulated by phosphorylation patterns. Phosphorylation at Ser5 inhibits beta strand formation while phosphorylation at Ser2 partially preserves beta propensity. This suggests that dynamic modulation of local CTD structure, rather than large-scale conformational changes, may be the key to how the CTD integrates phosphorylation signals to regulate interactions with binding partners throughout the transcription cycle.

## Experimental procedures

### Protein expression and purification

*CTD2′*. An *Escherichia coli* codon optimized DNA sequence encoding residues 1657 to 1739 of the *D. melanogaster* Rpb1 C-terminal domain (CTD2′) was cloned into the pET His6 MBP TEV LIC cloning vector (1M, Addgene plasmid #29656) using ligation independent cloning. This vector encodes a 6xHis-tagged MBP and a tobacco etch virus (TEV) protease cleavage sites at the N terminus of the CTD2′. After TEV cleavage, a cloning artifact (amino acids SNAG) remains at the N terminus of the CTD2′, which does not affect the biophysical properties of CTD2′, as they match those of a previous construct with a GPG cloning artifact (36). BL21 (DE3) cells containing the CTD2′ plasmid were grown in LB media with 50 μg/ml kanamycin at 37 °C and 200 rpm. At an optical density at 600 nm of 0.7 to 0.8, expression of was induced with 0.5 mM IPTG at 37 °C and 200 rpm for 3 h. Cells were pelleted by centrifugation at 5000*g* and stored at −80 °C. Cell pellets of CTD2′ were resuspended in the lysis buffer (50 mM Tris pH 7.5, 500 mM NaCl, 20 mM imidazole, and 2.5 mM β-mercaptoenthanol) supplemented with 1x EDTA-free protease inhibitor cocktail (Millipore), 1 mM PMSF, and 10 units of RNAse-free DNAse (NEB). Following sonication on ice, the cell lysate was centrifuged at 14000*g* for 45 min. The supernatant was passed through Ni-NTA resins (Thermo Fisher Scientific) equilibrated with the lysis buffer and washed with 10 column volumes of the lysis buffer. Proteins were eluted with the elution buffer (50 mM Tris pH 7.5, 500 mM NaCl, 200 mM imidazole, and 2.5 mM β-mercaptoenthanol). The eluate was incubated with TEV protease (1 mg per 1 L growth) under dialysis against 2 L of 50 mM Tris pH 7.5, 100 mM NaCl, and 2.5 mM β-mercaptoenthanol at 4 °C for 16 h. Dialysates were passed through a second Ni-NTA column, and the flow-through containing CTD2′ was collected and concentrated using a Amicon Ultra Centrifugal Filter with a 3 kDa molecular weight cutoff (MilliporeSigma). The concentration of CTD2′ was determined by A_280_ using the extinction coefficient ε^CTD2′^_280_ = 16,390 M^−1^ cm^−1^.

*Dyrk1a*. BL21 (DE3) cells were cotransformed with plasmids containing the kinase domain of human Dyrk1a (Addgene Plasmid #79690) and the lambda phosphatase (Addgene Plasmid #79748) (55). Cells were grown in LB media with 100 μg/ml ampicillin and 50 μg/ml spectinomycin at 37 °C and 200 rpm until the optical density at 600 nm reaches 0.6 to 0.8. Expression was induced with 0.2 mM IPTG at 18 °C and 200 rpm for 16 h. Cells were pelleted by centrifugation at 5000*g* and stored at −80 °C. Cell pellets of CTD2′ were resuspended in the lysis buffer (50 mM Tris pH 7.5, 500 mM NaCl, 20 mM imidazole, and 2.5 mM β-mercaptoenthanol) supplemented with 1x EDTA-free protease inhibitor cocktail (Millipore) and 1 mM PMSF. Following sonication on ice, the cell lysate was centrifuged at 14,000*g* for 45 min. The supernatant was passed through Ni-NTA resins (Thermo Fisher Scientific) equilibrated with the lysis buffer and washed with 10 column volumes of the lysis buffer. Proteins were eluted with the elution buffer (50 mM Tris pH 7.5, 500 mM NaCl, 200 mM imidazole, and 2.5 mM β-mercaptoenthanol). The eluate was incubated with TEV protease (1 mg per 1 L growth) to remove the 10xHis tag under dialysis against 2 L of 50 mM Tris pH 7.5, 100 mM NaCl, and 1 mM DTT at 4 °C for 16 h. The concentration of CTD2′ was determined by A_280_ using the extinction coefficient ε^Dyrk1a^_280_ = 50,310 M^−1^ cm^−1^.

### Kinase reactions

Phosphorylation time course reactions using Dyrk1a were performed with 2 ml of 46 μM CTD2′, 192 μl of 49 μM Dyrk1a, 10 mM ATP, 20 mM MgCl_2_, and 1 mM DTT at room temperature (21 °C). For MALDI-TOF analysis, 200 μl of the reaction mixture was taken at time points of 1, 2, 4, 6, and 24 h. Each sample was heated at 90 °C for 5 min to deactivate Dyrk1a.

### Matrix-assisted laser desorption/ionization-time of flight mass spectrometry

Samples of intact unphosphorylated and phosphorylated CTD2′ were desalted using Pierce C-18 Spin Columns (Thermo Fisher Scientific) and desiccated in a SpeedVac Vacuum Concentrator. The mass spectra were acquired on a Bruker Ultraflextreme MALDI TOF-TOF instrument using a factory-configured instrument method for the linear positive-ion detection over the 5000 to 20,000 *m/z* range. The sample-matrix mixture was prepared by combining one volume of 20 mg/ml super-DHB (Sigma p/n 50,862-1G-F) in 50% acetonitrile, 0.1% trifluoroacetic acid, 0.1% phosphoric acid with one volume of sample, 5 to 10 mg/ml in water, and 1 μl of this mixture was applied to a polished steel target. Laser power attenuation and pulsed ion extraction time were optimized to achieve the best signal. Instrument was calibrated using a Bruker Protein Mix I calibration standard containing a mixture of four proteins: bovine insulin, bovine ubiquitin, equine heart cytochrome C, and equine apomyoglobin. Mass spectra were smoothed (Savitzky–Golay, width 5 m/z, one cycle) and baseline subtracted (TopHat). Mass lists were generated using a centroid peak detection algorithm.

### Nano-liquid chromatography tandem mass spectrometry (Nano-LC MS^2^)

For alkylation and chymotrypsin digestion, 100 to 200 μg of phosphorylated CTD2′ in 100 mM Tris–HCl pH 8, 2 mM calcium chloride, and 10 mM DTT was incubated at 60 °C for 45 min to reduce cysteine residues. After cooling the sample to room temperature, iodoacetamide was added to a final concentration of 20 mM for cysteine alkylation. The reaction was incubated at room temperature for 30 min protected from light. The same amount of DTT as in the reduction step was added to quench the reaction. Briefly, 1 mg/ml of chymotrypsin endoproteinase, TLCK treated, MS Grade (Thermo Fisher Scientific) was added to a final 1:20 enzyme-to-protein sample ratio. The reaction was incubated at 37 °C for 16 to 24 h (overnight) and stored at −80 °C until MS analysis.

The chymotryptic peptides were separated on a Thermo Easy-nLC 1200 using 0.1% formic acid in water as mobile phase A and 80% aqueous acetonitrile, 0.1% formic acid as mobile phase B at a flow rate of 300 nl/min. The peptides, 1 to 2 μg were loaded onto an Acclaim PepMap100 trapping column (75 μm × 2 cm, C18, 3 μm, 100 Å, Thermo Fisher Scientific) using 5% B and separated on an Acclaim PepMap RSLC column (50 μm × 15 cm, C18, 2 μm, 100 Å, Thermo Fisher Scientific) with a 30-min 5%-25% B, followed by 10 min 25%-45% B. The column was then flushed with 90% B for 6 min. A Thermo Eclipse mass spectrometer was operated in a cycle time dependent mode (2 s) with the following data-dependent parameters: full FT MS scan at R 120,000 followed by R 15,000 MS^2^ scans with higher energy collision dissociation activation (30% normalized collision energy). Only the precursors with charge states 2 to 6 were selected for MS^2^; precursor isolation was in the quadrupole with a 1.6 *m/z* isolation window. The dynamic exclusion duration was set to 10 s.

The mass spectra were processed using the Proteome Discoverer 2.5 (Thermo Fisher Scientific) and searched with Sequest HT and MS Amanda 2.0 search engines and an IMP-ptmRS module for the phosphorylation analysis. The database contained the CTD2' and the Dyrk1a kinase sequences and 299 sequences of common contaminant proteins. The search parameters included precursor tolerance 10 ppm, fragment tolerance 0.02 Da, dynamic modifications included phosphorylation (+79.966 Da, S, T, Y) and oxidation (+15.995 Da, M), static modification was carbamidomethyl (+57.021 Da, C). Only peptides with a High Sequest HT confidence score were used for phosphorylation site assignment.

### NMR data collection and analysis

CTD2′ for NMR experiments was expressed in M9 minimal media with ^13^C and ^15^N enrichment achieved through the incorporation of ^15^N-ammonium chloride and ^13^C D-glucose (Cambridge Isotope Laboratories). The proteins were purified as described above. Phosphorylated CTD2′ was prepared by kinase reactions with 700 μl of 434 μM CTD2′ with a 12:1 M ratio of CTD2′ to Dyrk1a, with 10 mM ATP, 20 mM MgCl_2_, 1 mM DTT. The reaction mixture was dialyzed against 50 mM Tris pH 7.5, 100 mM NaCl, 10 mM ATP, 20 mM MgCl_2_, and 1 mM DTT. Reactions were incubated for 1 h and 48 h at room temperature (21 °C) to produce CTD2′ with 8 and 19 phosphorylation marks, respectively. Following incubation, samples were heated at 65 °C for 5 min and centrifuged to remove the Dyrk1a-containing pellets. The heating step did not affect the NMR spectra of CTD2′.

NMR data were collected on Bruker Avance III 600 MHz or Bruker Avance NEO 600 MHz spectrometers equipped with TCI triple-resonance cryoprobes. All data collection and initial processing were carried out in TopSpin (Bruker). ^13^C chemical shifts were referenced to sodium trimethylsilylpropanesulfonate (DSS). All NMR experiments were carried out in 80 mM imidazole pH 6.5, 50 mM KCl, 2.5 mM β-mercaptoethanol, and 10% D_2_O.

Chemical shift assignments of CTD2′ were carried out using ^13^C-Direct Detect methods (42). Assignments were generated based on CON experiments (8 scans, 1024 (^13^C) × 256 (^15^N) complex data points, with sweep widths of 18 ppm and 42 ppm, respectively) collected from ∼1 mM ^13^C, ^15^N, CTD2′ samples. Three-dimensional experiments for assignments included the following: (HACA)N(CA)CON (8 scans, 1024 (^13^C) × 64 (^15^N) × 128 (^15^N), with sweep widths of 12 ppm, 42 ppm, and 42 ppm, respectively); (HACA)N(CA)NCO (8 scans, 1024 (^13^C) × 64 (^15^N) × 128 (^15^N), with sweep widths of 12 ppm, 42 ppm, and 42 ppm, respectively); CCCON (8 scans, 1024 (^13^C) × 64 (^15^N) × 128 (^13^C), with sweep widths of 12 ppm, 42 ppm, and 80 ppm, respectively). Peak picking and assignments were carried out manually using NMRFAM-Sparky. Average chemical shift perturbations were calculated as follows:

$$\Delta \delta_{AV}={\left\{\left[{0.4\left(\Delta \delta_{N}\right)}^{2}+{\left(\Delta \delta_{C^{\prime }}\right)}^{2}\right]\right\}}^{1/2}$$

Random coil values of unphosphorylated and phosphorylated CTD2′ at 25 °C and pH 6.5 were obtained from the Kragelund database (https://www1.bio.ku.dk/english/research/bms/sbinlab/randomchemicalshifts2/).

### Size-exclusion chromatography with multiangle light scattering

After purification as described above, CTD2′ was further purified using size-exclusion chromatography with P-10 resins in 80 mM imidazole pH 6.5, 50 mM KCl, and 1 mM DTT. The sample was then concentrated to 1.6 mM. Subsequently, 20 μl of CTD2′ was loaded onto a Superdex S-100 Increase column (Cytiva) equilibrated with 25 mM sodium phosphate pH 7.5, 50 mM NaCl, and 4 mM DTT.

SEC–MALS experiment was conducted using an Agilent 1260 Infinity II HPLC system equipped with an autosampler all set to room temperature. Wyatt Technology DAWN MALS, Wyatt Optilab refractive index detector, and Agilent UV detector were used for analyzing the molar mass of peaks that eluted from the column. The SEC–MALS system was equilibrated for 5 h with buffer consisting of 25 mM sodium phosphate pH 7.5, 50 mM NaCl, and 4 mM DTT. UV was set to 280 nm and temperature was set to 4 °C. A volume of 20 μl of sample was injected at a flow rate of 0.5 ml min^−1^ with a chromatogram run time of 48 min. The sample concentration was 10 mg/ml. Dynamic light scattering data were collected using an inline Wyatt Dynapro Nanostar equipment.

Normalization and alignment of the MALS and refractive index detectors were carried out on standard bovine serum albumin. The concentration source used was the refractive index. Data were analyzed using the ASTRA software Version 8.2.2 (Wyatt). The chromatogram showed a single peak in the light scattering and the refractive index that was monodisperse. Molar mass (Mw) showed a mass consistent with a monomer = 8.8 kDa. The hydrodynamic radius was estimated to be 16.12 (±1.761%) Å, where the error is from fitting.

### Small-angle X-ray scattering

SAXS experiments were performed using a Rigaku MM007 rotating anode X-ray source paired with the BioSAXS2000nano Kratky camera system. This setup includes OptiSAXS confocal max-flux optics, designed specifically for SAXS, and a highly sensitive HyPix-3000 Hybrid Photon Counting detector. The sample-to-detector distance was calibrated to 495.5 mm using silver behenate powder (The Gem Dugout, State College, PA). The momentum transfer scattering vector (q-space) range, defined as 4πsin(θ)/λ, extended from qmin = 0.008 Å^−1^ to qmax = 0.6 Å^−1^ (where q = 4πsin(θ)/λ and 2θ is the scattering angle). The X-ray beam energy was set to 1.2 keV, with a Kratky block attenuation of 23% and a beam diameter of approximately 100 μm. For sample handling, protein samples were loaded into a quartz capillary flow cell using the Rigaku autosampler, with the sample stage maintained at room temperature. The entire X-ray flight path, including the beam stop, was held under vacuum conditions of < 1 × 10^−3^ torr to reduce air scattering. Automated data collection, including detailed cleaning cycles between samples, was managed by the Rigaku SAXSLAB software.

After purification as described above, samples of CTD2′ for SAXS were further purified using size-exclusion chromatography with P-10 resins in 80 mM imidazole pH 6.5, 50 mM KCl, and 1 mM DTT. Phosphorylation reactions were set up with a 60:1 M ratio of CTD2′ to Dyrk1a, in the presence of 10 mM ATP, 20 mM MgCl_2_, and 1 mM DTT at 37 °C for 40 min, followed by incubation at room temperature (21 °C) for 48 h. Phosphorylation of CTD2′ was confirmed by MALDI-TOF mass spectrometry. Dyr1ka was removed using Q Sepharose HP chromatography in 25 mM Tris pH 7.5 and 1 mM DTT with a KCl gradient from 50 mM to 1 M. The eluted phosphorylated CTD2′ was then buffer exchanged into 25 mM Tris pH 7.5, 50 mM KCl, and 1 mM DTT. For SAXS, two sample concentrations were used: 5 mg/ml and 9.4 mg/ml for unphosphorylated CTD2′, and 3 mg/ml and 4.3 mg/ml for Dyrk1a phosphorylated CTD2′. We also performed SAXS on the SEC-MALS eluted fraction of unphosphorylated CTD2′, which was at a concentration of 2 mg/ml.

Data processing was completed using the Rigaku SAXSLAB software, with each dataset averaged from six ten-minute images across three replicates of both protein and buffer samples to confirm the absence of X-ray radiation damage. Consistency in SAXS data overlays verified no radiation decay or sample loss over the 60-min collection period. Reference buffer subtraction was subsequently performed to isolate the protein's raw SAXS scattering curve. The forward scattering I(0) and radius of gyration (Rg) were calculated using the Guinier approximation, which assumes the intensity follows I(q) = I(0)exp[−1/3(qRg)^2^] at very small angles (q < 1.3/Rg). Data analysis included evaluating parameters such as radius of gyration, D_max_, Guinier fits, Kratky plots, and the pair distance distribution function with ATSAS software. The pairwise distance distribution function P(r) was generated using GNOM following protocols for IDPs (56, 57), where D_max_ was increased until P(r) smoothly approaches zero at larger r.

## Data availability

NMR assignments for unphosphorylated and Dry1ka-phosphorylated (8P and 19P) CTD2′ have been deposited in the BMRB database (entry numbers: 52794, 52795, and 52796). Other data have been deposited in the Penn State ScholarSphere (58) (https://scholarsphere.psu.edu/resources/1ff42a7c-4d1e-4c3e-8313-dd50b849cf1d).

## Supporting information

This article contains supporting information.

## Conflict of interest

The authors declare that they have no conflicts of interest with the contents of this article.
