# Supplementary material for: Phosphorylation modulates secondary structure of intrinsically disorder regions in RNA polymerase II
Source: J Biol Chem. 2025 Apr 22;301(6):108533. doi: 10.1016/j.jbc.2025.108533 (PMC12152636; doi:10.1016/j.jbc.2025.108533)
Supplement: Supporting information [file mmc1.docx]

**Supporting Information for**

Phosphorylation modulates secondary structure of intrinsically disorder regions in RNA polymerase II

Wei Chen (陳瑋)^1,2^, Tatiana N. Laremore^3^, Neela H. Yennawar^3^, and Scott A. Showalter^1,2,a)^

^1^Department of Chemistry, The Pennsylvania State University, University Park, Pennsylvania 16802, USA

^2^Center for Eukaryotic Gene Regulation, Department of Biochemistry and Molecular Biology, The Pennsylvania State University, University Park, Pennsylvania 16802, USA

^3^Huck Institutes of the Life Sciences, The Pennsylvania State University, University Park, Pennsylvania 16802, USA

a) Author to whom correspondence should be addressed: sas76@psu.edu

**This PDF file includes:**

Figures S1 to S9

Table S1

| 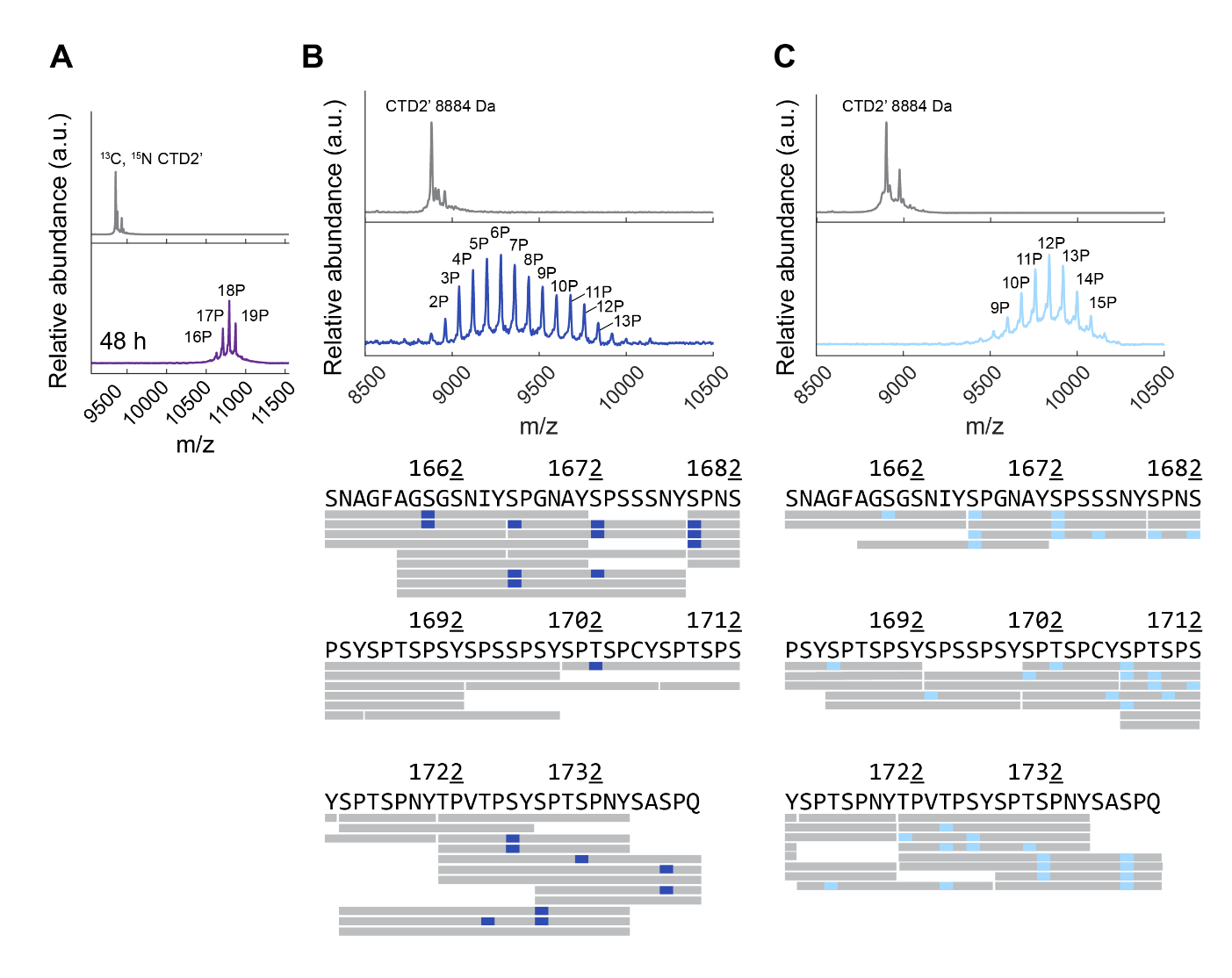 |
| --- |
| **Figure S1.** **Phosphorylation of CTD2’ by Dyrk1a characterized with mass spectrometry.** **(A)** MALTI-TOF mass spectra of unphosphorylated and Dyrk1a phosphorylated CTD2’ after 48 hours of incubation, showing a maximum of 19 phosphorylation events. No additional phosphorylation marks were observed compared to the 24-hour sample. **(B, C)** MALDI-TOF mass spectra of Dyrk1a phosphorylated CTD2’ with an average number of 6 and 12 phosphorylation marks, and the corresponding sequence coverage of chymotrypsin digested peptides showing phosphorylation sites identified by LC-MS2. |

| 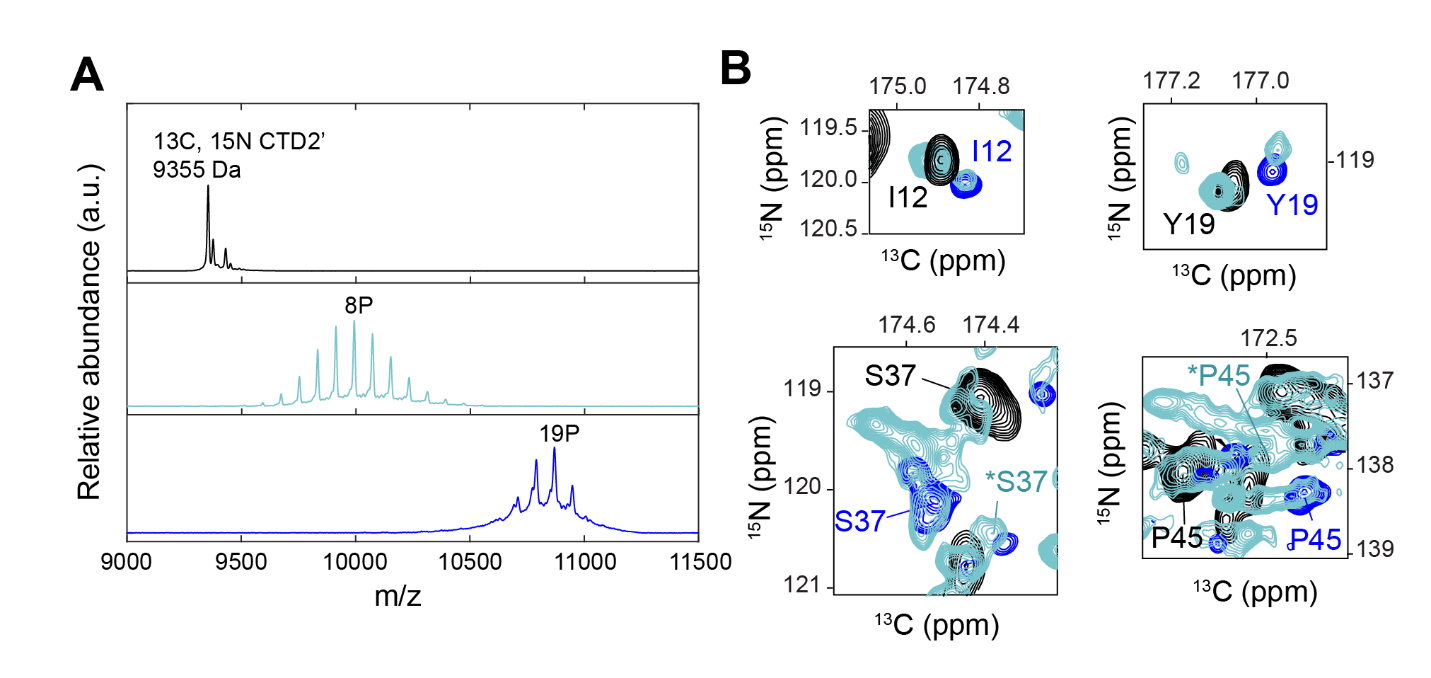 |
| --- |
| **Figure S2. NMR on Dyrk1a phosphorylated CTD2’. (A)** MALDI-TOF mass spectra of unphosphorylated, and two Dyrk1a phosphorylated ^13^C, ^15^N CTD2’ NMR samples. **(B)** Representative peaks from CON spectra showing Dyrk1a phosphorylated CTD2’ 8P (cyan) overlapping with unphosphorylated CTD2’ (black) and hyperphosphorylated CTD2’ 19P (blue), as well as unique peaks for Dyrk1a phosphorylated CTD2’ 8P denoted by asterisks (*). |


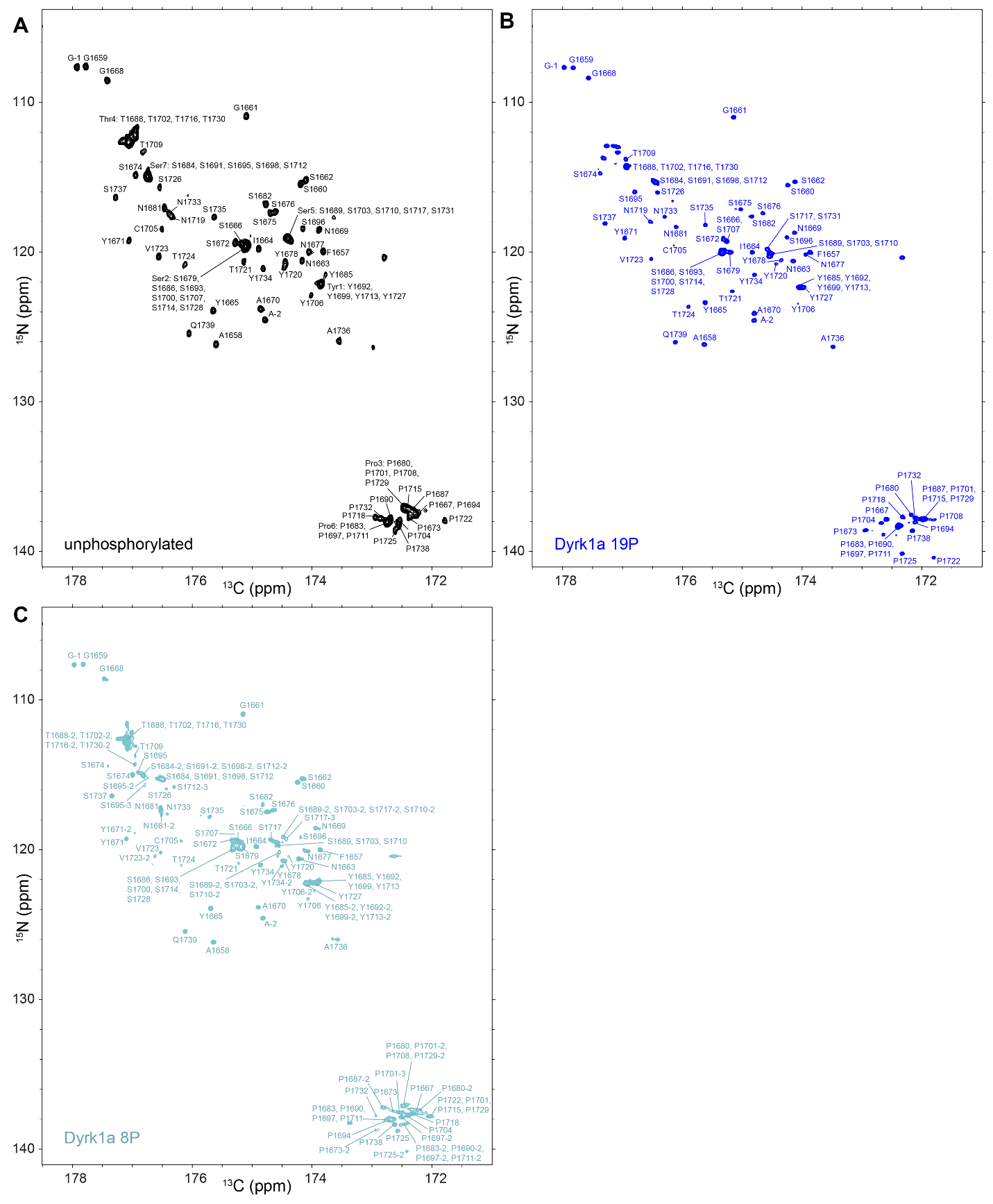


**Figure S3. Assigned ^13^C, ^15^N-CON spectra of CTD2’, (A**) Unphosphorylated. **(B)** Dyrk1a phosphorylated 19P. **(C)** Dyrk1a phosphorylated 8P.


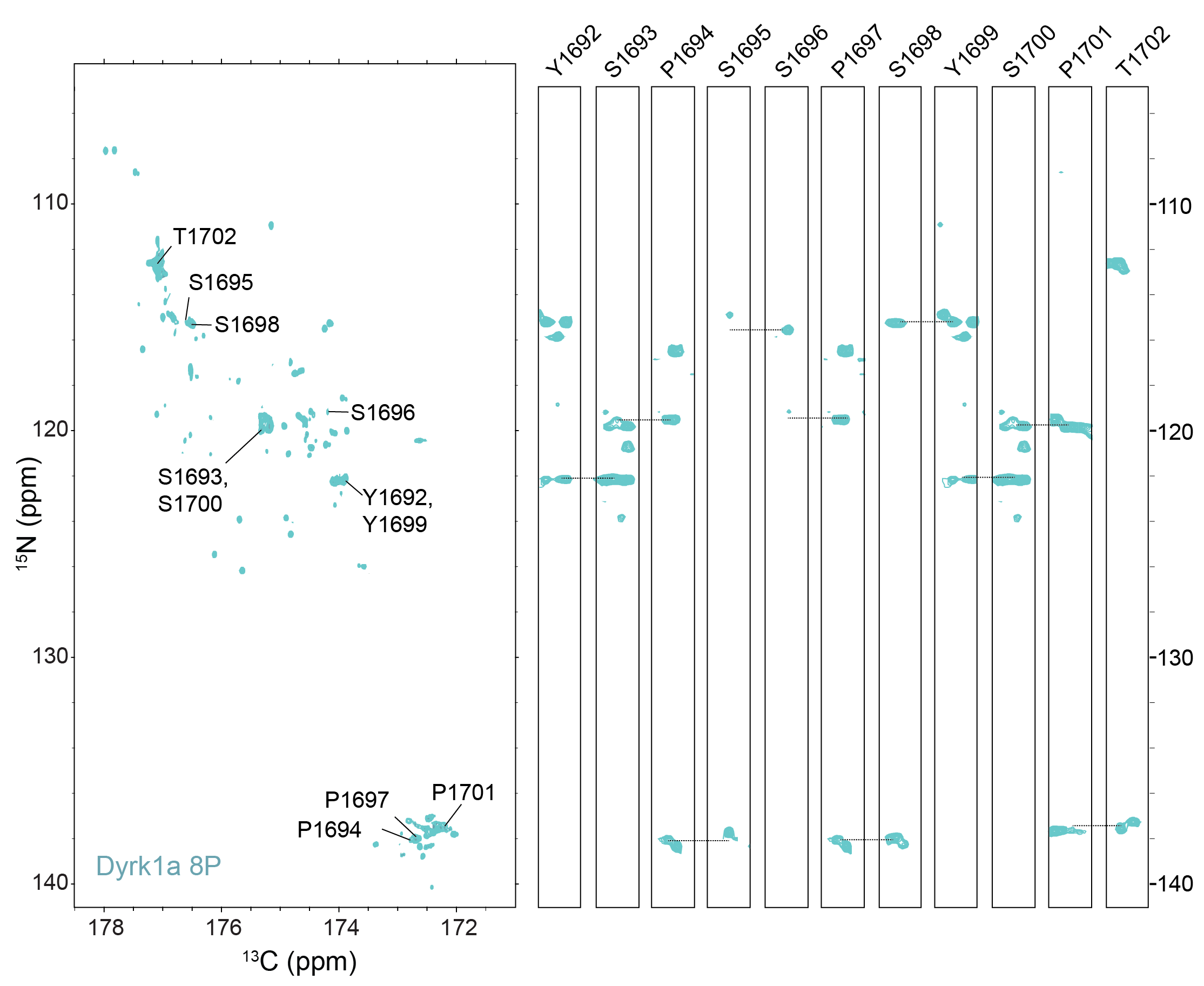


**Figure S4. Example of backbone assignment using ^13^C direct-detect NMR.** The 3D (HACA)N(CA)CON experiment provides backbone connectivity. In the 2D ^13^C, ^15^N-CON spectrum (left), residues Y1692 to T1702 are labeled. Each residue has a corresponding (HACA)N(CA)CON strip (right), which links ^15^N (i) to ^15^N (i-1). This example illustrates how uniquely assigned residues, such as S1696 and P1697, are connected to ‘clusters’ of residues (Tyr1 cluster: Y1692, Y1699, Ser2 cluster: S1693, S1700). We note that for S1695 and S1696, the ^15^N (i) ‘self peaks’ in the strips are not visible. However, this does not affect assignment, as the process relies on the 15N (i-1) peaks.


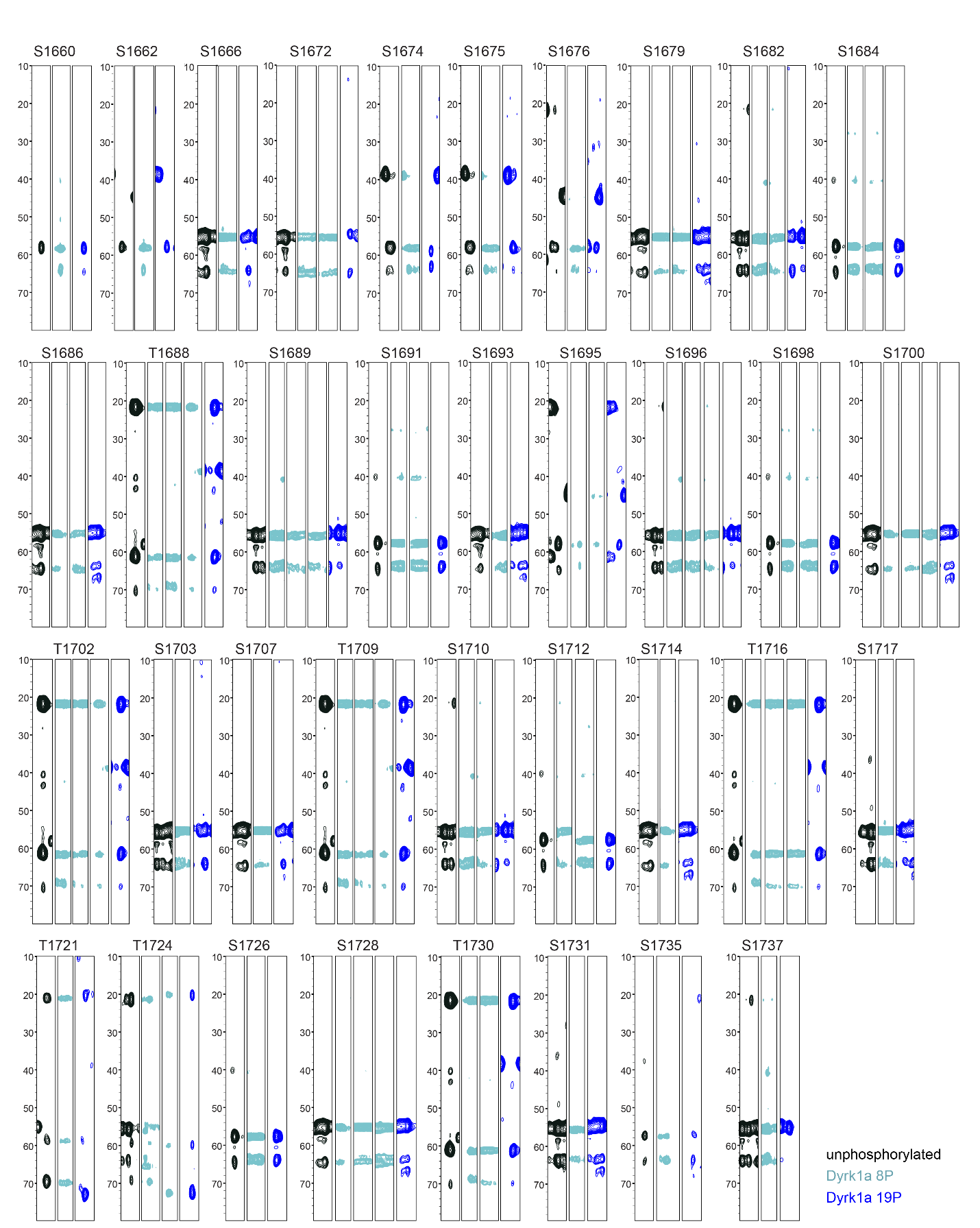
**Figure S5.** **CCCON strips for aliphatic ^13^C resonances**. All states for Dyrk1a phosphorylated CTD2’ 8P are included. Notably, phosphorylation caused a ~ 2 ppm shift in C_β_ resonances (~70 ppm) for T1721 and T1724 for Dyrk1a 19P. The shift was also observed in a minor state for T1724 for Dyrk1a 8P.

| 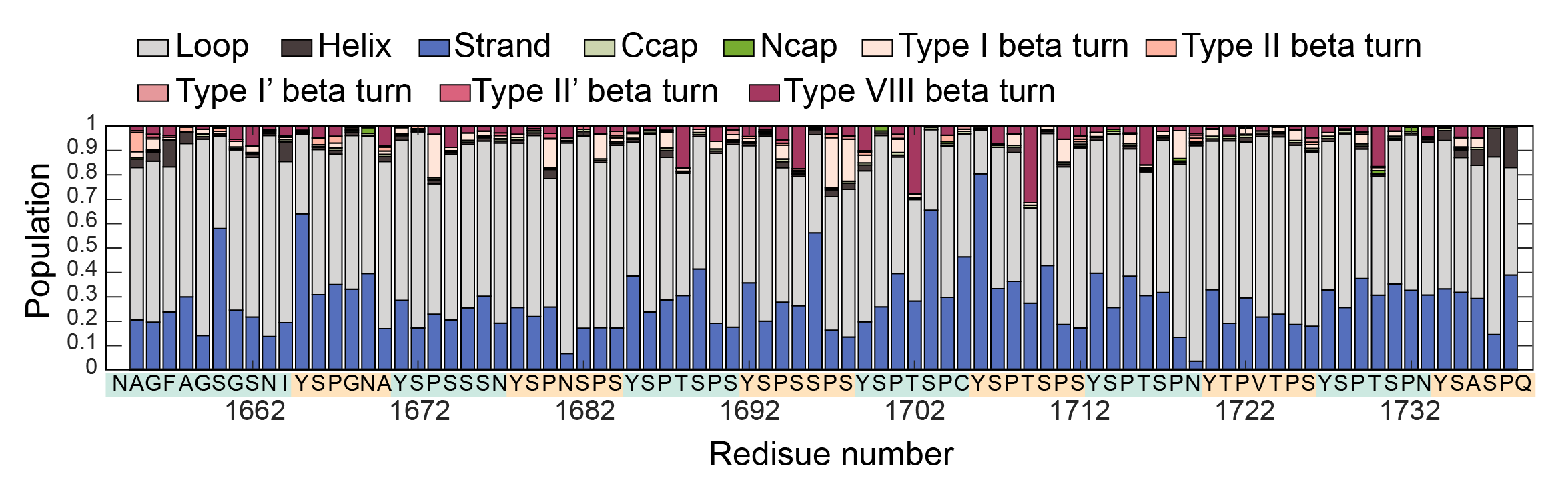 |
| --- |
| **Figure S6. Predicted structural motif populations in unphosphorylated CTD2’ using the MICS (Motif Identification from Chemical Shifts) program**. Populations of loops, helices, strands, N-terminal and C-terminal helix capping motifs (Ncap and Ccap), and five types of beta-turns (I, II, I’, II’, and VIII) were calculated based on backbone and sidechain chemical shifts. |

| 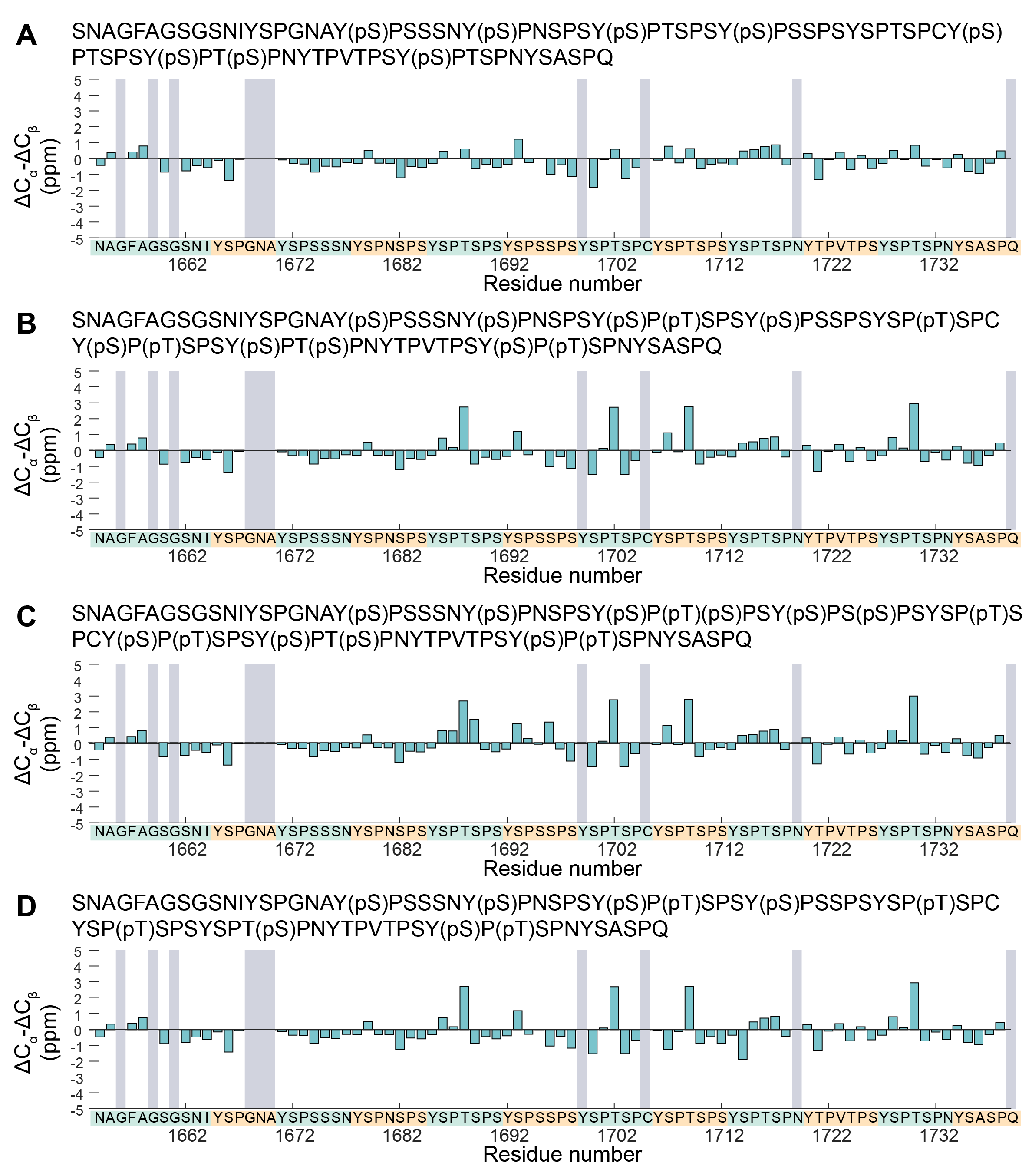 |
| --- |
| **Figure S7. Secondary chemical shifts for Dyrk1a phosphorylated CTD2’ (8P) (A–D)** Secondary chemical shifts generated using four different random coil reference sets from different phosphorylation assignments. Residues without available C_α_ and/or C_β_ chemical shift values are indicated by grey areas. |

| 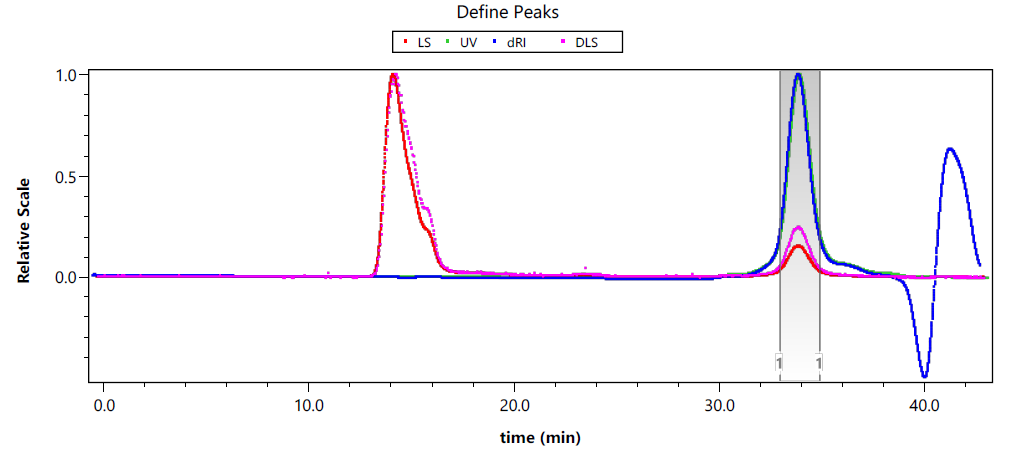 |
| --- |
| **Figure S8. SEC-MALS on CTD2’.** CTD2’ eluted at ~34 min as a monomer with a molecular weight of 8845 (±1.507%) Da, consistent with its theoretical molecular weight 8848 Da. |

| 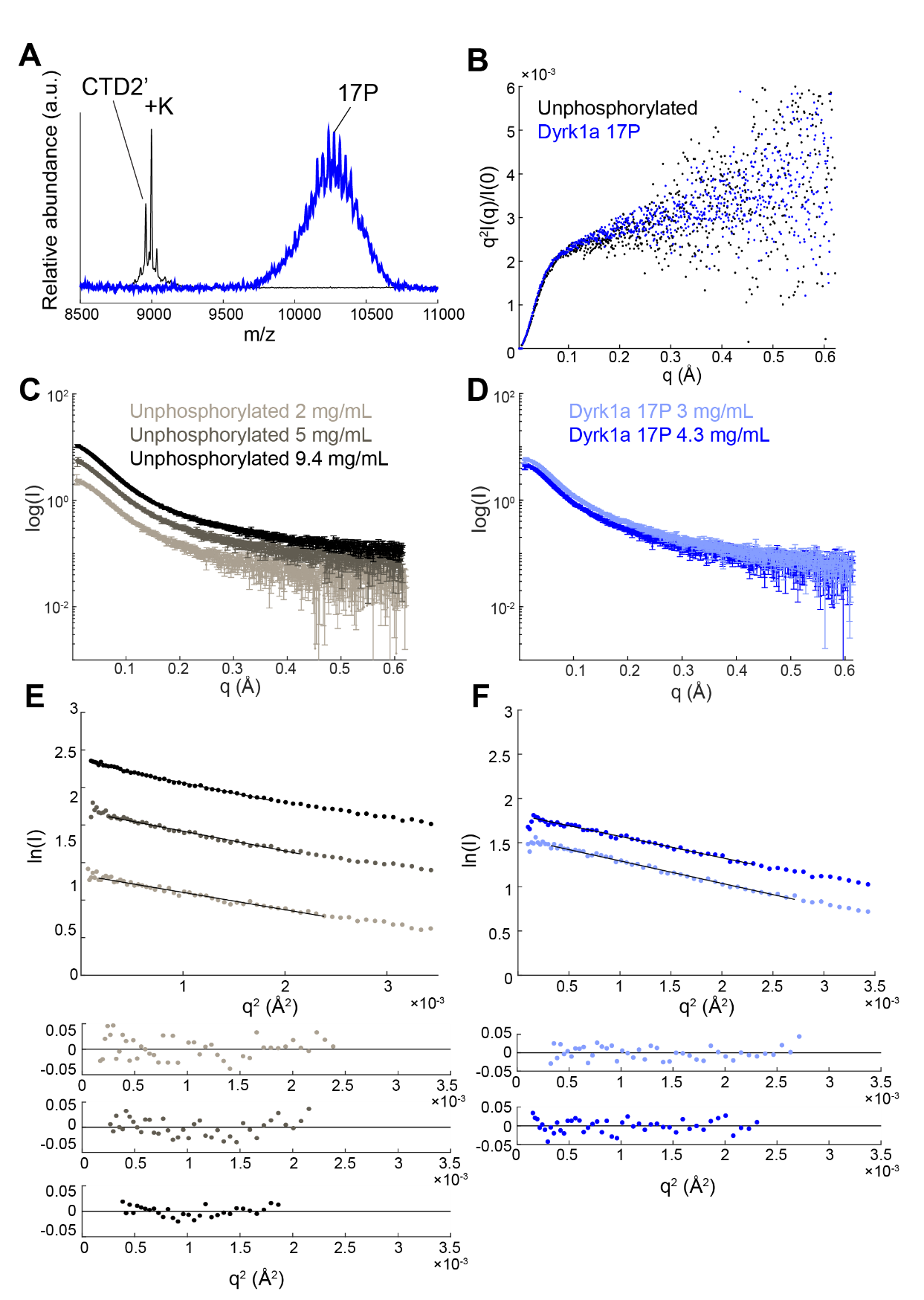 |
| --- |
| **Figure S9. SAXS on unphosphorylated and Dyrk1a phosphorylated CTD2’.** **(A)** MALDI-TOF mass spectra showing the SAXS sample of Dyrk1a phosphorylated CTD2’ contained an average of 17 phosphorylation marks. **(B)** Kratky plots for unphosphorylated and Dyrk1a phosphorylated CTD2’. **(C)** Raw scattering data for unphosphorylated CTD2’. **(D)** Raw scattering data for Dyrk1a phosphorylated CTD2’. **(E)** Guinier fitting and residuals for unphosphorylated CTD2’. **(F)** Guinier fitting and residuals for Dyrk1a phosphorylated CTD2’. |

**Table S1. SAXS analysis on unphosphorylated and Dyr1ka phosphorylated CTD2’**

| **Unphosphorylated CTD2’** | | | | | | |
| --- | --- | --- | --- | --- | --- | --- |
| Concentration (mg/mL) | R_g_ (Å) Guinier | Range (points) | I(0) | R_g_ (Å) from P(r) | Range (points) | D_max_ (Å) |
| 2 | 26.13 ± 0.31 | 13-57 | 2.29 ± 0.017 | 28.42 ± 2.34 | 13-368 | 115 |
| 5 | 27.99 ± 0.28 | 17-54 | 5.36 ± 0.035 | 33.55 ± 5.67 | 17-341 | 135 |
| 9.4 | 28.48 ± 0.22 | 21-50 | 10.28 ± 0.052 | 31.58 ± 10.56 | 21-335 | 120 |
| **Dyrk1a phosphorylated CTD2’** | | | | | | |
| Concentration (mg/mL) | R_g_ (Å) Guinier | Range (points) | I(0) | R_g_ (Å) from P(r) | Range (points) | D_max_ (Å) |
| 3 | 28.24 ± 0.25 | 11-53 | 4.76 ± 0.027 | 31.49 ± 4.49 | 11-337 | 128 |
| 4.3 | 26.88 ± 0.22 | 12-56 | 6.12 ± 0.032 | 28.95 ± 6.26 | 12-355 | 110 |
